# Green Synthesis and Catalytic Activity of Gold Nanoparticles Synthesized by *Artemisia capillaris* Water Extract

**DOI:** 10.1186/s11671-016-1694-0

**Published:** 2016-10-26

**Authors:** Soo Hyeon Lim, Eun-Young Ahn, Youmie Park

**Affiliations:** College of Pharmacy and Inje Institute of Pharmaceutical Sciences and Research, Inje University, 197 Inje-ro, Gimhae, Gyeongnam 50834 Republic of Korea

**Keywords:** Gold nanoparticles, Catalytic activity, 4-Nitrophenol reduction reaction, *Artemisia capillaris* extract

## Abstract

Gold nanoparticles were synthesized using a water extract of *Artemisia capillaris* (AC-AuNPs) under different extract concentrations, and their catalytic activity was evaluated in a 4-nitrophenol reduction reaction in the presence of sodium borohydride. The AC-AuNPs showed violet or wine colors with characteristic surface plasmon resonance bands at 534~543 nm that were dependent on the extract concentration. Spherical nanoparticles with an average size of 16.88 ± 5.47~29.93 ± 9.80 nm were observed by transmission electron microscopy. A blue shift in the maximum surface plasmon resonance was observed with increasing extract concentration. The face-centered cubic structure of AC-AuNPs was confirmed by high-resolution X-ray diffraction analysis. Based on phytochemical screening and Fourier transform infrared spectra, flavonoids, phenolic compounds, and amino acids present in the extract contributed to the reduction of Au ions to AC-AuNPs. The average size of the AC-AuNPs decreased as the extract concentration during the synthesis was increased. Higher 4-nitrophenol reduction reaction rate constants were observed for smaller sizes. The extract in the AC-AuNPs was removed by centrifugation to investigate the effect of the extract in the reduction reaction. Interestingly, the removal of extracts greatly enhanced their catalytic activity by up to 50.4 %. The proposed experimental method, which uses simple centrifugation, can be applied to other metallic nanoparticles that are green synthesized with plant extracts to enhance their catalytic activity.

## Background

With the development of nanotechnology, metallic nanoparticles (MNPs) have attracted considerable attention due to their wide range of applications. Among MNPs, gold nanoparticles (AuNPs) have been considered an interesting area of research for useful applications in drug/gene delivery, catalysis, sensing, and imaging [1, 2]. Studies of AuNPs regarding synthesis, electrochemistry, and optical properties have been well reviewed by Sardar and co-workers [3]. AuNPs were first used in catalysis for the oxidation of carbon monoxide, in which AuNPs were supported on a transition metal oxide [4]. Examples of organic reactions catalyzed by AuNPs include the following [5]: (i) hydrogenation reactions: hydrogenation of unsaturated carbonyls and reduction of nitro groups; (ii) alkyne activation; (iii) coupling reactions; (iv) oxidation reactions: oxidation of cyclohexane, oxidation of toluene, oxidation of alcohols, and oxidation of alkenes; and (v) miscellaneous reactions. The use of AuNPs in catalysis is advantageous. One of the merits of AuNPs is their high surface-area-to-volume ratio, which allows for the enhancement of chemical reactivity. The reduction of 4-nitrophenol (4-NP) in the presence of excess sodium borohydride is the most frequently used model reaction for evaluating the catalytic activity of AuNPs. The reaction product, 4-aminophenol (4-AP), is an important intermediate for analgesics and antipyretics.

The most common method for synthesizing AuNPs involves chemical reducing agents for the reduction of Au ions to AuNPs. However, these chemical methods employ toxic reagents, and the resulting AuNPs are most likely unsuitable for in vitro and in vivo applications. These applications require processes that are green, environmentally benign, and eco-friendly. Recent sustainability initiatives have proposed the use of plant extracts as reducing agents in the synthesis of AuNPs. The use of plant extracts possesses several advantages. (i) The reaction is facile and can be performed in a single pot; (ii) the reaction is easy to scale up; (iii) the resulting AuNPs are biocompatible; and (iv) synergistic activities are expected due to the combination of two materials (i.e., plant extracts and AuNPs). Many studies have reported the green synthesis of AuNPs using the following plant extracts as reducing agents and catalysts in the 4-NP reduction reaction: *Bupleurum falcatum* [6], *Salicornia brachiata* [7], *Aerva lanata* [8], *Coleus forskohlii* [9], *Phoenix dactylifera* [10], *Garcinia combogia* [11], *Trigonella foenum*-*graecum* [12], *Gnidia glauca* [13], and *Breynia rhamnoides* [14].

The aerial part of *Artemisia capillaris* (Compositae) has been used in traditional Chinese medicine and displays antiviral, antibacterial, and anti-inflammatory activities [15]. Our laboratory has reported the synthesis of silver nanoparticles (AgNPs) using *A. capillaris* extract as a reducing agent [16, 17]. The synthesized AgNPs showed excellent antibacterial activity against Gram-negative bacteria [16]. Furthermore, AgNPs synthesized using *A. capillaris* in the presence of cetyltrimethylammonium bromide exhibited antibacterial properties against methicillin-resistant *Staphylococcus aureus* [17].

In the present study, *A. capillaris* water extract was used as a reducing agent to synthesize AuNPs (hereafter referred to as AC-AuNPs). The catalytic activity of AC-AuNPs was then evaluated in the 4-NP reduction reaction in the presence of excess sodium borohydride. To investigate the effect of extract concentration on the catalytic activity, five different extract concentrations were used to synthesize AC-AuNPs. Additionally, the extract was removed by centrifugation (referred to hereafter as *cf*-AC-AuNPs), and the catalytic activities of AC-AuNPs and *cf*-AC-AuNPs were compared in the reduction reaction of 4-NP to 4-AP.

## Methods

### Materials

Hydrochloroauric acid trihydrate (HAuCl_4_·3H_2_O), 4-nitrophenol, and sodium borohydride were purchased from Sigma-Aldrich (St. Louis, MO, USA). *A. capillaris* was purchased from Ominherb (Uiseong-gun, Gyeongsangbuk-do, Republic of Korea). All other reagents were of analytical grade. Syringe filters (0.45 μm) were purchased from Sartorius Stedim Biotech (Goettingen, Germany). All solutions were prepared in deionized water.

### Instruments

A Shimadzu UV-2600 was used to acquire UV-visible spectra with a quartz cuvette (Shimadzu Corporation, Kyoto, Japan). A JEM-2100F microscope operated at 200 kV was used for transmission electron microscopy (TEM) imaging (JEOL Ltd., Tokyo, Japan). The nanoparticle solution was loaded onto a carbon-coated copper grid (carbon type B, 300 mesh, Ted Pella Inc., Redding, CA, USA), and the sample-loaded grid was dried for 24 h at ambient temperature prior to TEM analysis. Hydrodynamic size and zeta potential measurements were performed using a NanoBrook 90Plus Zeta (Brookhaven Instruments Corporation, Holtsville, NY, USA). Each sample was measured ten times to determine the hydrodynamic size and five times to determine the zeta potential; the measured values were then averaged to obtain mean values. High-resolution X-ray diffraction (HR-XRD) was performed at 2*θ* values ranging from 20° to 90° using a Bruker D8 Discover high-resolution X-ray diffractometer equipped with a Cu-Kα radiation source (*λ* = 0.154056 nm) (Bruker, Karlsruhe, Germany). FT-IR spectra were acquired using a Varian 640IR in the attenuated total reflectance mode (Agilent Technologies, Santa Clara, CA, USA). A FD8518 freeze dryer was used to prepare powdered samples (IlShinBioBase Co. Ltd., Gyeonggi-do, Republic of Korea). Centrifugation was performed using either a 5424R (Eppendorf AG, Hamburg, Germany) or UNION 55R (Hanil Science Industrial Co. Ltd., Incheon, Republic of Korea).

### Preparation of *A. capillaris* Water Extract

**Fig. 1:**
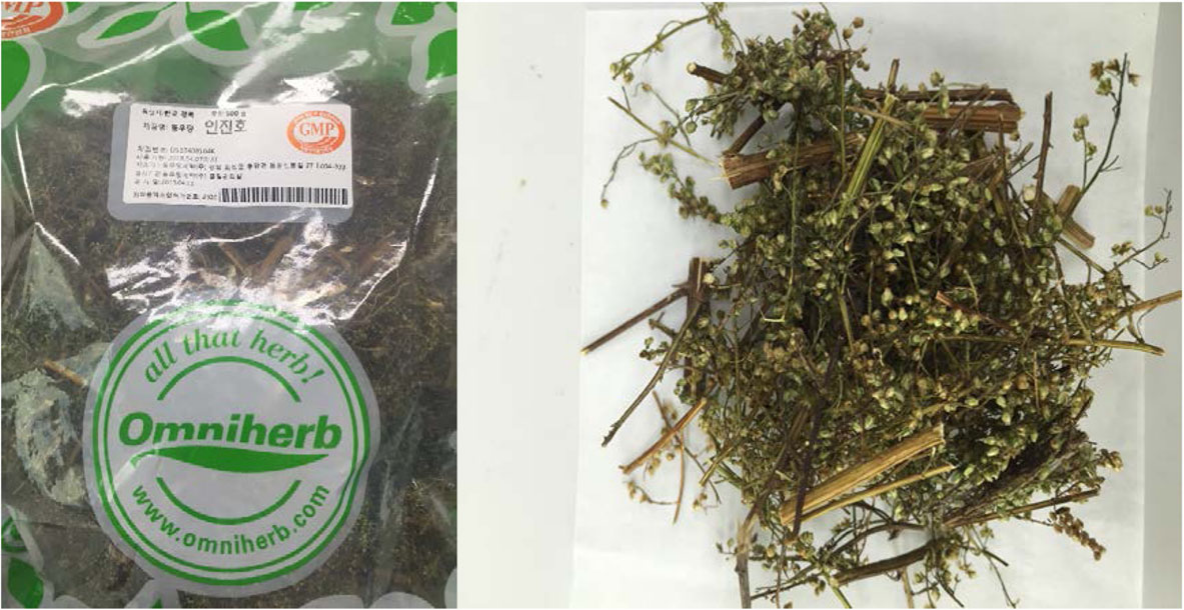
Digital photographs of *A. capillaris* that was used in the current report

Digital photographs of the aerial part of *A. capillaris* used in the current study are shown in Fig. 1. *A. capillaris* was pulverized, and the pulverized powder was mixed with deionized water in a ratio of 10:90 (the pulverized powder: deionized water, *v*/*v*). The extraction process was then performed by sonication for 3 h. After sonication, the supernatant was pooled. This step was repeated three times. The pooled supernatant was filtered using filter paper. The filtrate was then freeze-dried and stored at −80 °C in a freezer. To prepare the extract stock solution, the freeze-dried powder was dissolved in deionized water. Then, centrifugation (UNION 55R) was conducted at 22 °C (30 min, 7,546*g*). The supernatant was taken and syringe-filtered (0.45 μm). The filtrate was freeze-dried, and the freeze-dried powder was labeled “*A. capillaris* water extract” and dissolved in deionized water to a final concentration of 0.1 % (*w*/*v*). This solution was used as the stock solution for the synthesis of AuNPs and phytochemical screening. For phytochemical screening (Table 1), 20 tests were conducted according to the procedures reported in previous studies [18, 19]. The detailed procedures of the following six tests are described in reference [19]: tannic acid test, ninhydrin test, general glycosides test, hydroxyanthraquinone test, Keller-Killiani test, and Baljet’s test. The other tests were performed according to the procedures described in reference [18].

**Table 1:** Phytochemical screening of *A. capillaris* water extract

Alkaloids	Mayer’s test	−
	Wagner’s test	−
	Hager’s test	−
	Tannic acid test	−
Amino acids	Ninhydrin test	+
Carbohydrates	Molish’s test	−
	Fehling’s test	−
Saponins	Froth test	++
Phenolic compounds	Gelatin test	−
	Ferric chloride test	+
Flavonoids	Alkaline reagent test	+
	Lead acetate test	+
Glycosides	General test	−
Anthraquinone glycosides	Modified Borntrager’s test	−
	Hydroxyanthraquinone test	−
Cardiac glycosides	Baljet’s test	−
	Keller-Killiani test	−
Phytosterols	Liebermann-Burchard’s test	−
	Salkowski’s test	−
Diterpenes	Copper acetate test	+

^+^Fairly present

^++^Highly present

−Absent

### Synthesis of AC-AuNPs

Two stock solutions with concentrations of 0.1 % (extract) and 1 mM (hydrochloroauric acid trihydrate) were prepared in deionized water. For the synthesis of AC-AuNPs, the final concentration of hydrochloroauric acid trihydrate was fixed at 0.25 mM. The extract concentration was varied at 0.015, 0.025, 0.035, 0.045, and 0.055 %. The final volume was adjusted to 4 mL by adding deionized water. The incubation was conducted at 80 °C in a dry oven for 1 h. UV-visible spectra were acquired between 400 and 700 nm, and the hydrodynamic size and zeta potentials were measured.

### Preparation of *cf*-AC-AuNPs

Centrifugation was performed to remove the extract from AC-AuNPs (1 mL) using a 5424R centrifuge at 24 °C (18,000*g*, 20 min). After centrifugation, the supernatant containing the extract was removed, and the pellet was re-dispersed with deionized water (final volume 1 mL) to generate *cf*-AC-AuNPs. UV-visible spectra were acquired between 400 and 700 nm, and the hydrodynamic size and zeta potentials were measured.

### Catalytic Activity of AuNP Catalysts in 4-NP Reduction Reaction

Stock solutions of sodium borohydride and 4-NP were freshly prepared prior to use. 4-NP (0.4 mM, 1 mL) and sodium borohydride (40 mM, 1 mL) were mixed in a 4 mL quartz cuvette. Then, 100 μL of either AC-AuNPs or *cf*-AC-AuNPs was added, and the final volume was adjusted to 4 mL with deionized water. Upon the addition of AuNP catalysts, the reaction progress was monitored every 5 min by UV-visible spectrophotometry between 200 and 500 nm range at 25 °C. In order to evaluate the reusability of catalysts, *cf*-AC-AuNPs was centrifuged at 24 °C (18,000*g*, 20 min), and the pellet was re-dispersed with deionized water (final volume 1 mL). Also, the catalytic activity was assessed in the presence of sodium borohydride with the same procedure described above.

## Results and Discussion

### Synthesis of AC-AuNPs

**Fig. 2:**
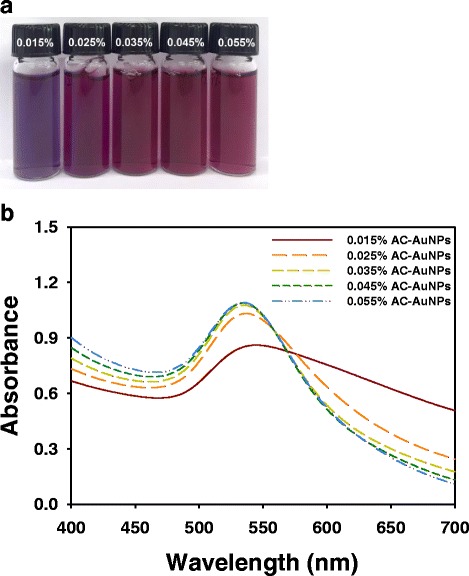
AC-AuNPs. **a** Digital images and **b** UV-visible spectra of AC-AuNPs which were synthesized with five different extract concentrations

UV-visible spectrophotometry is the most convenient tool for determining the success of synthesis. Owing to the surface plasmon resonance (SPR) of AuNPs, the color of the AC-AuNPs solution was violet (0.015 %) or wine (0.025~0.055 %) which was dependent on the extract concentration (Fig. 2a). The SPR values of AC-AuNPs at each extract concentration were as follows: 543 nm (0.015 %), 537 nm (0.025 %), 535 nm (0.035 %), 534 nm (0.045 %), and 534 nm (0.055 %). Thus, the SPR showed a tendency to blue shift with increasing extract concentration.

### Preparation of *cf*-AC-AuNPs

**Fig. 3:**
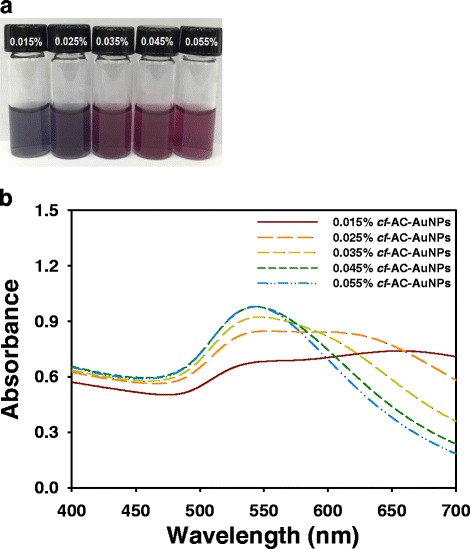
*cf*-AC-AuNPs. **a** Digital images and **b** UV-visible spectra of *cf*-AC-AuNPs which were prepared by centrifugation of AC-AuNPs. The detailed procedure is in the experimental section

As previously mentioned, to compare the catalytic activities of AC-AuNPs and *cf*-AC-AuNPs, the extract was removed from AC-AuNPs to produce *cf*-AC-AuNPs. Centrifugation is the simplest approach for removing extracts from AC-AuNPs. After centrifugation, the supernatant containing the extracts was removed, and the pellet was re-dispersed with water. As shown in Fig. 3a, the color of the solutions changed from violet to gray (0.015 %) and from wine to violet (0.025 %). For the three other concentrations (0.035, 0.045, and 0.055 %), the wine color of the *cf*-AC-AuNPs remained the same. Centrifugation led to the broadening of the UV-visible spectra (Fig. 3b), indicating the presence of polydisperse *cf*-AC-AuNPs. *cf*-AC-AuNPs showed broad SPR when prepared under extract concentrations of 0.015 and 0.025 %. New SPR signals emerged for *cf*-AC-AuNPs near 600~650 nm at these two concentrations. When the extract concentration was increased from 0.035 to 0.055 %, the SPR at 600~650 nm tended to disappear.

### TEM Images and Size Histograms

**Fig. 4:**
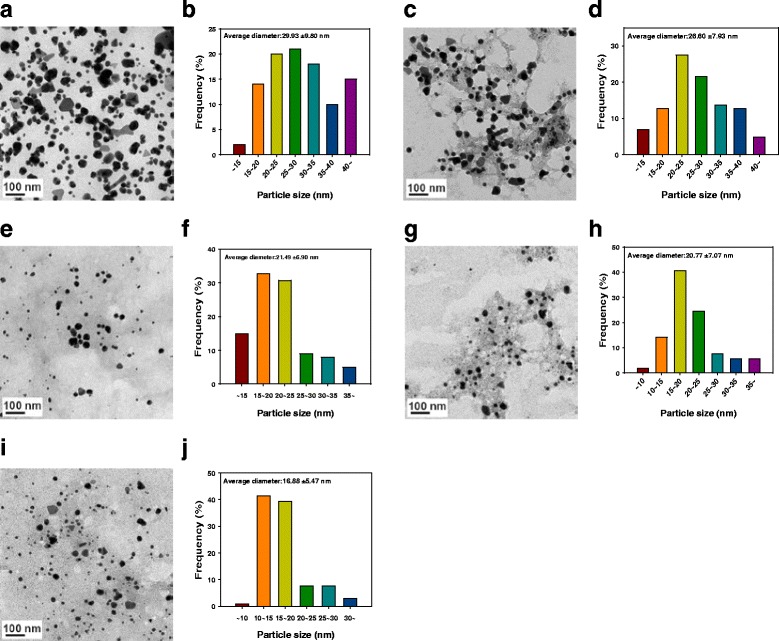
TEM images and size histograms of AC-AuNPs. **a**, **b** 0.015 %. **c**, **d** 0.025 %. **e**, **f** 0.035 %. **g**, **h** 0.045 %. **i**, **j** 0.055 %. The *scale bar* represents 100 nm in each image. Randomly selected nanoparticles in TEM images were used for constructing size histograms. Numbers of discrete nanoparticles selected from each images were **a** 100, **c** 102, **e** 101, **g** 106, and **i** 104

The synthesized AC-AuNPs were mostly spherical, as shown in Fig. 4. Minor fractions of particles showing other shapes, including triangles and rods, were also observed. More than 100 nanoparticles were randomly selected in TEM images obtained for each extract concentration to plot size histograms. The numbers of nanoparticles selected from the TEM images were as follows: 100 nanoparticles for 0.015 %, 102 nanoparticles for 0.025 %, 101 nanoparticles for 0.035 %, 106 nanoparticles for 0.045 %, and 104 nanoparticles for 0.055 %. The average size of the AC-AuNPs was measured to be 29.93 ± 9.80 nm for 0.015 %, 26.60 ± 7.93 nm for 0.025 %, 21.49 ± 6.90 nm for 0.035 %, 20.77 ± 7.07 nm for 0.045 %, and 16.88 ± 5.47 nm for 0.055 %. As the extract concentration was increased, the average size of the AC-AuNPs decreased. The size histograms showed a Gaussian distribution. Using a high extract concentration for synthesis caused strong binding between the surface of the AuNPs and extracts. The organic components of the extract may act as capping agents preventing nanoparticle growth. Therefore, a high concentration of extract leads to a reduction in nanoparticle size. The same results were reported in the work of Naraginiti and Sivakumar, in which *C. forskohlii* root extract was utilized as a reducing agent [9].

### Hydrodynamic Size and Zeta Potential Measurements

In aqueous medium, the hydrodynamic size can be measured by dynamic light scattering. Generally, the hydrodynamic size is larger than that measured from nanoparticles in TEM images. With an increase in the extract concentration of AC-AuNPs, the hydrodynamic size also increased from 26.9 to 41.3 nm (Table 2). The obtained result was the opposite of that observed for the size measured from TEM images discussed in the previous section. Using the TEM images, only the size of the metallic cores was measured, whereas the hydrodynamic size reflects the size of metallic cores and the extracts on the surface of AuNPs. Therefore, the higher concentration of the extract contributed to the larger hydrodynamic size. By contrast, the hydrodynamic size of *cf*-AC-AuNPs decreased from 91.1 to 55.2 nm with an increase in extract concentration.

**Table 2:** Hydrodynamic size (*n* = 10) and zeta potential (*n* = 5) values of AC-AuNPs and *cf*-AC-AuNPs

	*A. capillaris* final concentration (%)	0.015	0.025	0.035	0.045	0.055
AC-AuNPs	Hydrodynamic size (nm) (polydispersity index)	26.9 (0.358)	29.0 (0.353)	33.9 (0.328)	37.1 (0.308)	41.3 (0.286)
	Zeta potential (mV)	−16.45	−19.97	−18.13	−20.92	−20.63
*cf*-AC-AuNPs	Hydrodynamic size (nm) (polydispersity index)	91.1 (0.325)	67.1 (0.325)	58.8 (0.309)	56.9 (0.306)	55.2 (0.296)
	Zeta potential (mV)	−29.17	−27.15	−29.56	−30.19	−30.27

To predict the colloidal stability, zeta potential measurements are generally performed. As shown in Table 2, negative zeta potentials were observed for the AC-AuNPs (−16.45~−20.63 mV). With an increase in extract concentration, the absolute value of the zeta potential increased. This result demonstrated that the colloidal stability of the AC-AuNPs was improved by increasing the extract concentration. Negative values were also observed for the *cf*-AC-AuNPs (−27.15~−30.27 mV); however, the zeta potential values of the *cf*-AC-AuNPs showed no general tendency.

### HR-XRD Analysis

**Fig. 5:**
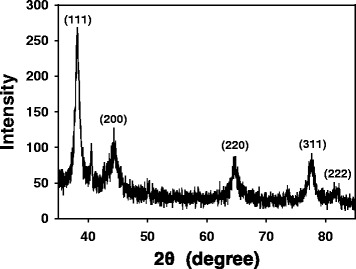
HR-XRD analysis of AC-AuNPs that were synthesized with 0.035 % extract concentration

The crystalline nature of AuNPs is commonly confirmed by HR-XRD analysis. As shown in Fig. 5, the AC-AuNPs (0.035 %) showed distinct diffraction peaks at 38.22°, 44.12°, 65.22°, 77.64°, and 82.24°. These peaks correspond to the (111), (200), (220), (311), and (222) planes of crystalline Au, respectively. The (111) peak was the most intense among these, suggesting that the (111) plane was the dominant plane in the crystalline Au structures.

### Phytochemical Screening and FT-IR Spectra

A series of phytochemical screenings was conducted to verify the presence of primary and secondary metabolites in the *A. capillaris* extract. As shown in Table 1, saponins, amino acids, phenolic compounds, flavonoids, and diterpenes were present in the extract.

**Fig. 6:**
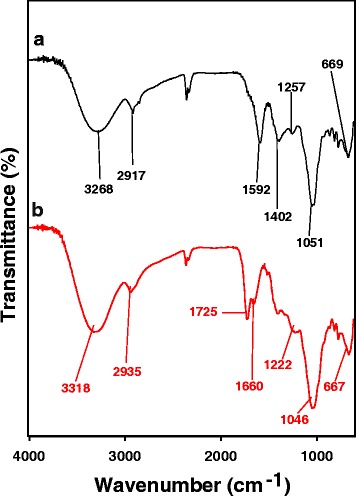
FT-IR spectra of **a** *A. capillaris* water extract and **b** AC-AuNPs that were synthesized with 0.035 % extract concentration

FT-IR spectra are commonly recorded to identify the functional groups of the compounds that contribute to the reduction of Au ions to AuNPs. The FT-IR spectra of the extract and AC-AuNPs are shown in Fig. 6. The FT-IR spectrum of the extract shows peaks at 3268, 2917, 1592, 1402, 1257, 1051, and 669 cm^−1^, whereas that of the AC-AuNPs shows peaks at 3318, 2935, 1725, 1660, 1222, 1046, and 667 cm^−1^. The characteristic hydroxyl group was observed in the extract at 3268 cm^−1^ [13]. This peak was shifted to 3318 cm^−1^ in AC-AuNPs, suggesting that hydroxyl functional groups were involved in the synthesis of AC-AuNPs. This result is well correlated with the phytochemical screening results presented in Table 1, which indicate that flavonoids and phenolic compounds were present as secondary metabolites. A peak associated with the C–H stretching of alkanes appeared at 2917 cm^−1^ in the spectrum of the extract, and this peak was shifted to 2935 cm^−1^ after the synthesis of AC-AuNPs. Furthermore, two peaks were found at 1660 and 1046 cm^−1^ in AC-AuNPs, corresponding to amide I and C–N stretching vibrations, respectively. The appearance of these two peaks in AC-AuNPs implied that amino acids/proteins played a role in reducing Au ions to AuNPs and their complexation (or adsorption) onto the surface of AuNPs. Amino acids were present in the extract, as indicated by the phytochemical screening, which corroborates the FT-IR results. Interestingly, the appearance of the peak at 1725 cm^−1^ in the AC-AuNP spectrum indicated that carbonyl functional groups also affected the reduction of Au ions. Gangula and co-workers have reported that flavonones and terpenoids possibly bind to the surface of AuNPs via an interaction through carbonyl groups or π electrons [14]. Thus, the appearance of carbonyl functional groups at 1725 cm^−1^ in AC-AuNPs demonstrated that flavonoids could bind to the surface of AC-AuNPs. Collectively, the phytochemical screening and FT-IR results suggest that the flavonoids, phenolic compounds, and amino acids present in the extract are mostly likely involved in the reduction of Au ions and/or are complexed (or adsorbed) on the surface of AC-AuNPs.

### Catalytic Activity of AC-AuNPs and *cf*-AC-AuNPs in 4-NP Reduction Reaction

**Fig. 7:**
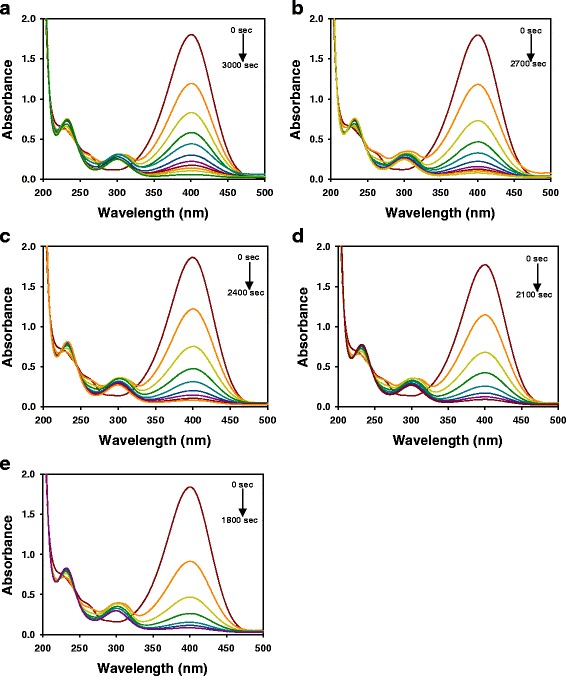
UV-visible spectra of 4-NP reduction reaction with AC-AuNP catalysts. AC-AuNPs were synthesized with extract concentrations of **a** 0.015 %, **b** 0.025 %, **c** 0.035 %, **d** 0.045 %, and **e** 0.055 %

**Fig. 8:**
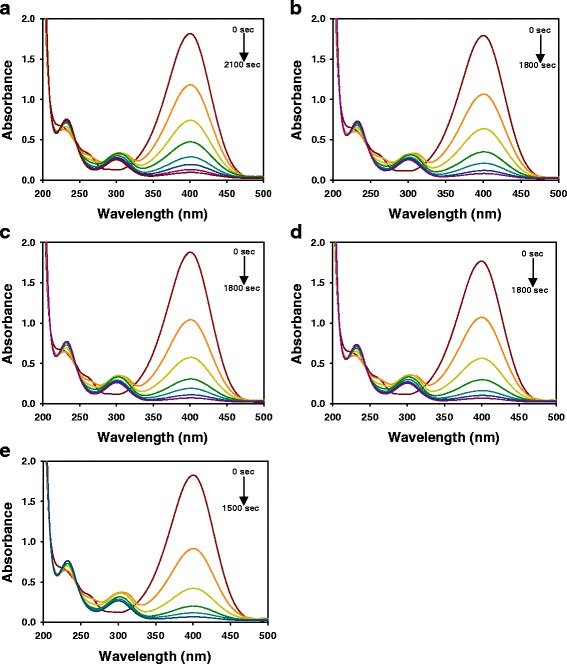
UV-visible spectra of 4-NP reduction reaction with *cf*-AC-AuNP catalysts. *cf*-AC-AuNPs were prepared by centrifugation of AC-AuNPs. The detailed procedure is in the experimental section. **a** 0.015 %. **b** 0.025 %. **c** 0.035 %. **d** 0.045 %. **e** 0.055 %

As previously mentioned, the 4-NP reduction reaction is generally used as a model reaction for evaluating the catalytic activity of AuNPs. One of the merits of using the 4-NP reduction reaction is that it is easy to follow the reaction progress by UV-visible spectrophotometry. An excess amount (100-fold) of sodium borohydride relative to the concentration of 4-NP was used to ensure pseudo-first-order kinetics. In the presence of sodium borohydride, 4-NP forms a 4-nitrophenolate ion that absorbs at 400 nm. Without the addition of AuNP catalysts, the abundance of 4-nitrophenolate ion does not change. As either AC-AuNPs or *cf*-AC-AuNPs were added, the absorbance at 400 nm began to decrease (Figs. 7 and 8). Simultaneously, an absorbance peak appeared at 300 nm due to the reaction product 4-AP. As shown in Fig. 7, the extract concentration of AC-AuNPs affected the time required for the completion of the reaction. The rate of the reaction increased with the extract concentration (Fig. 7). The times required for reaction completion were as follows: 3000 s for 0.015 %, 2700 s for 0.025 %, 2400 s for 0.035 %, 2100 s for 0.045 %, and 1800 s for 0.055 %. In the case of *cf*-AC-AuNPs, the reaction rate also increased with extract concentration (Fig. 8). However, the time required for reaction completion was shorter than that for AC-AuNPs.

**Fig. 9:**
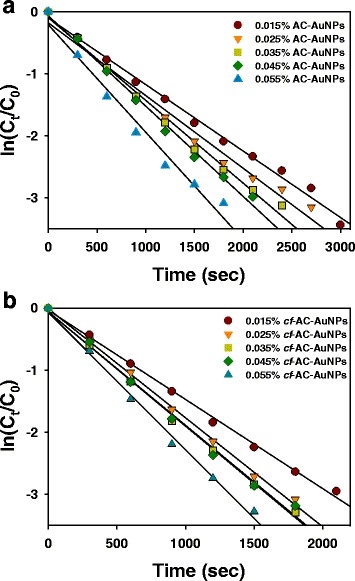
**a**, **b** The relationship between ln(*C*_*t*_/*C*_0_) and time (sec) of 4-NP reduction reaction with either AC-AuNP or *cf*-AC-AuNP catalysts in the presence of excess sodium borohydride. AC-AuNPs were synthesized with different extract concentrations. *cf*-AC-AuNPs were prepared by centrifugation of AC-AuNPs. The detailed procedure is in the experimental section. (*circle*) 0.015 %, (*inverted triangle*) 0.025 %, (*square*) 0.035 %, (*diamond*) 0.045 %, (*triangle*) 0.055 %

The relationship between ln(*C*_*t*_/*C*_*0*_) and time (sec) is shown in Fig. 9. *C*_*t*_ and *C*_*0*_ are the concentrations of 4-NP at time *t* and *0*, respectively. The linear relationship of AC-AuNPs and *cf*-AC-AuNPs demonstrates that the reaction followed pseudo-first-order kinetics. The rate constant (*k*) was obtained from the slope of the linear equation as follows.

−*kt* = ln(*C*_*t*_/*C*_0_) = ln(*A*_*t*_/*A*_0_), where *A*_*t*_ and *A*_0_ are substituted for *C*_*t*_ and *C*_0_, respectively. *A*_*t*_ and *A*_0_ are the absorbance of 4-NP at 400 nm at time *t* and time 0, respectively.

The reaction rate increased with the extract concentration used for the synthesis of AC-AuNPs (Fig. 9a). The same results were obtained for *cf*-AC-AuNPs (Fig. 9b). The rate constants of both AC-AuNPs and *cf*-AC-AuNPs for the 4-NP reduction reaction are summarized in Table 3. Under all extract concentrations, the rate constants of *cf*-AC-AuNPs (1.44 × 10^−3^~2.21 × 10^−3^ s^−1^) were higher than those of AC-AuNPs (1.07 × 10^−3^~1.73 × 10^−3^ s^−1^). The rate constant increased by 27.7~50.4 % for *cf*-AC-AuNPs. Thus, it is noteworthy that the removal of the extracts by a simple centrifugation process enhanced the rate constants of *cf*-AC-AuNPs in the 4-NP reduction reaction. Furthermore, the rate constants for smaller nanoparticles were higher than those for larger AuNPs. The same result was observed by Zayed and Eisa, who synthesized AuNPs with *P. dactylifera* leaf extract [10]. As shown in Table 3, *cf*-AC-AuNPs could be reusable on the first recycle; however, the rate constants were much lower (0.038 ×10^-3^~1.75 ×10^-3^s^−1^) than those of *cf*-AC-AuNPs. An attempt was made for the reuse of *cf*-AC-AuNPs on the second recycle; however, the catalytic reaction did not proceed any further.

**Table 3:** Rate constants in 4-NP reduction reaction with AuNP catalysts in the presence of sodium borohydride

	*A. capillaris* final concentration (%)	0.015	0.025	0.035	0.045	0.055
Rate constant (sec^−1^)	AC-AuNPs	1.07 × 10^−3^	1.17 × 10^−3^	1.33 × 10^−3^	1.46 × 10^−3^	1.73 × 10^−3^
	*cf*-AC-AuNPs (percentage of increase compared to AC-AuNPs)	1.44 × 10^−3^ (34.6 %)	1.76 × 10^−3^ (50.4 %)	1.84 × 10^−3^ (38.3 %)	1.87 × 10^−3^ (28.1 %)	2.21 × 10^−3^ (27.7 %)
	1st recycle of *cf*-AC-AuNPs	0.038 × 10^−3^	0.038 × 10^−3^	0.39 × 10^−3^	1.75 × 10^−3^	0.75 × 10^−3^

## Conclusions

Plant extracts are promising reducing agents for the synthesis of AuNPs in fulfilling global sustainability initiatives. In the current study, an *A. capillaris* extract was successfully utilized as a reducing agent for the synthesis of AC-AuNPs under different extract concentrations. Phytochemical screening indicated that saponins, amino acids, phenolic compounds, flavonoids, and diterpenes were present in the *A. capillaris* water extract. Among these compounds, flavonoids, phenolic compounds, and amino acids were involved in the synthesis, as verified by FT-IR spectroscopy. The average size of AC-AuNPs decreased with an increase in extract concentration, as measured by TEM imaging. The extract was then removed from AC-AuNPs to produce *cf*-AC-AuNPs, and the catalytic activities of both sets of nanoparticles in the 4-NP reduction reaction were compared. Interestingly, the rate constants increased with increasing extract concentration for both AC-AuNPs and *cf*-AC-AuNPs. These results imply that the metallic core size of AuNPs mostly likely affects their catalytic activities. Small nanoparticles possess a large surface-area-to-volume ratio, increasing their catalytic activities. Finally, the removal of the extract enhanced the particles’ catalytic activity by up to 50.4 %. Thus, the current method, which uses simple centrifugation, can be applied to other metallic nanoparticles that are green-synthesized with plant extracts to enhance their catalytic activity in the 4-NP reduction reaction.
